# Mode and site of action of therapies targeting CGRP signaling

**DOI:** 10.1186/s10194-023-01644-8

**Published:** 2023-09-11

**Authors:** Alejandro Labastida-Ramírez, Edoardo Caronna, Cédric Gollion, Emily Stanyer, Austeja Dapkute, Diana Braniste, Hoda Naghshineh, Liga Meksa, Nino Chkhitunidze, Tamari Gudadze, Patricia Pozo-Rosich, Rami Burstein, Jan Hoffmann

**Affiliations:** 1 Wolfson Centre for Age-Related Diseases, Institute of Psychiatry, Psychology & Neuroscience, King’s College London, London, SE1 1UL UK; 2 Headache Unit, Neurology Department, Vall d’Hebron Universitary Hospital, Barcelona, Spain; 3 Headache Research Group, Vall d’Hebron Institut de Recerca, Universitat Autònoma de Barcelona, Barcelona, Spain; 4 Department of Neurology, University Hospital of Toulouse, Toulouse, France; 5 Nuffield Department of Clinical Neurosciences, Sleep and Circadian Neuroscience Institute, University of Oxford, Oxford, UK; 6 Centre of Neurology, Vilnius University, Vilnius, Lithuania; 7 Institute of Neurology and Neurosurgery, Diomid Gherman, Chișinău, Moldova; 8 State University of Medicine and Pharmacy, Nicolae Testemițanu, Moldova; 9 Headache Department, Iranian Center of Neurological Research, Neuroscience Institute, Tehran University of Medical Science, Tehran, Iran; 10 Headache Unit, Neurology and Neurosurgery Department, Riga East University Hospital Gailezers, Riga, Latvia; 11 Department of Neurology, Caucasus Medical Centre, Tbilisi, Georgia; 12 Department of Neurology, Christian Hospital Unna, Unna, Germany; 13 Department of Anesthesia, Critical Care and Pain Medicine, Beth Israel Deaconess Medical Center, Boston, MA USA; 14 Department of Anesthesia, Harvard Medical School, Boston, MA USA; 15 Center for Life Science, Room 649, 3 Blackfan Circle, Boston, MA 02215 USA; 16 NIHR-Wellcome Trust King’s Clinical Research Facility/SLaM Biomedical Research Centre, King’s College Hospital, London, UK

**Keywords:** Amylin, Anti-CGRP, Calcitonin, Headache, Migraine, Trigeminal

## Abstract

Targeting CGRP has proved to be efficacious, tolerable, and safe to treat migraine; however, many patients with migraine do not benefit from drugs that antagonize the CGRPergic system. Therefore, this review focuses on summarizing the general pharmacology of the different types of treatments currently available, which target directly or indirectly the CGRP receptor or its ligand. Moreover, the latest evidence regarding the selectivity and site of action of CGRP small molecule antagonists (gepants) and monoclonal antibodies is critically discussed. Finally, the reasons behind non-responders to anti-CGRP drugs and rationale for combining and/or switching between these therapies are addressed.

## Role of calcitonin gene-related peptide in nociceptive transmission

Calcitonin gene-related peptide (CGRP) is one of the most investigated molecules in headache pathogenesis. In 1982, a novel 37 amino acid neuropeptide was discovered that is derived from the alternative splicing of calcitonin gene (*CALCA*) mRNA in neural tissue [1]. It was hence named CGRP, later identified as α-CGRP. This peptide has an amphiphilic α-helix between residues 8–18 that is important in the interaction with CGRP receptors [2], which were found to be of multiple subtypes [3, 4]. CGRP is found in two isoforms in humans: α-CGRP and β-CGRP [5], the latter being encoded by a different *CALCB* gene, both expressing in the enteric nervous system [6] and the central nervous system (CNS) (reviewed in [7]). However, considering that that only α-CGRP plays a role in sensory trigeminal afferents and trigeminal pain-mediating areas in the CNS, and that for these reasons most studies focus on α-CGRP, this review will be limited to this isoform, with a focus on migraine and the trigeminovascular system.

**Fig. 1:**
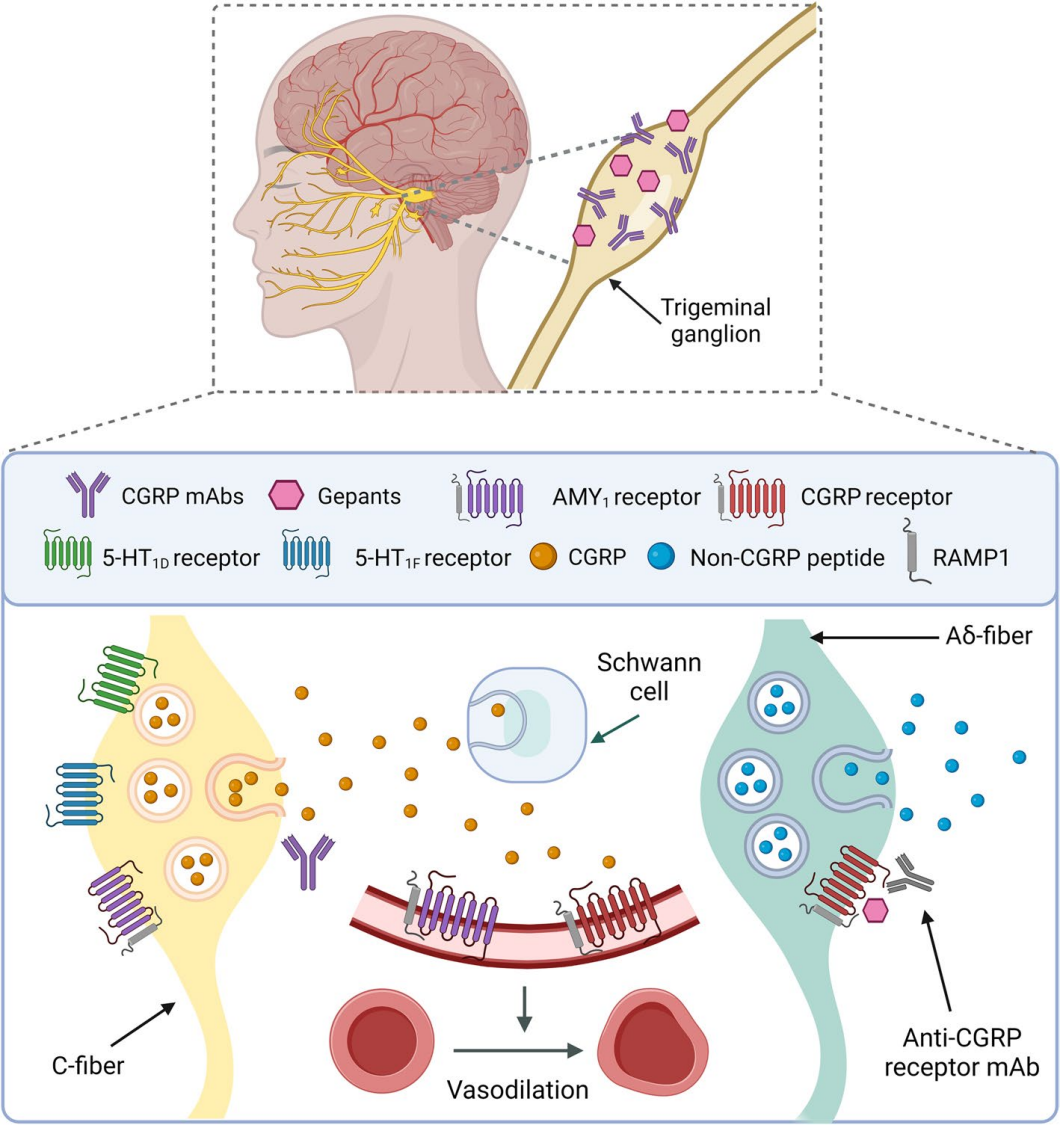
Anti-CGRP drugs and their peripheral sites of action in the trigeminovascular system. Schematic of CGRP-based therapies highlighting where the CGRP monoclonal antibodies (mAbs), CGRP receptor mAbs, and CGRP receptor antagonists (gepants) have their main site of action. The expression of AMY_1_ receptors remains to be fully determined. Adapted from [12, 21]

Although the exact mechanisms underlying the onset of a migraine attack remain to be determined, it is now well-established that the onset of the throbbing headache of migraine is mediated by CGRP release from the trigeminovascular system [8, 9]: a functional pathway consisting of sensory (pseudounipolar) neurons peripherally innervating the cranial meninges and their associated vasculature, whose cell somas are in the trigeminal ganglion (Fig. 1), and centrally projecting axons to the trigeminocervical complex that transmit nociceptive signals to the thalamus and higher order cortical regions [10–12]. Immunohistochemical studies have shown that CGRP is highly expressed in sensory unmyelinated C-fibers arising from the trigeminal ganglia and dorsal root ganglia (DRG) as well as their terminals in the spinal cord and brainstem [13, 14], with distribution correlating with CGRP binding site localization [15]. For instance, around 50% of human trigeminal ganglion (TG) neurons show CGRP-immunoreactivity [16]. Similarly, a recent mRNA study found that up to 60% of human DRG neuron express CGRP [17]. Moreover, rodent data has revealed that in comparison to the TG, CGRP mRNA levels are 20x – 250x lower in CNS structures such as lateral medulla and midbrain/hypothalamus, respectively [13]. CGRP released from trigeminal fibers located in the dura mater is unlikely to cross the blood–brain barrier (BBB) due to molecular size [18] and limited diffusion [19, 20].

CGRP was additionally shown to be located around isolated dural and cerebral arteries, where it produces vasodilation [22, 23]. Vasodilatory properties of CGRP were found to be one of the most potent identified in humans [24]. In 1987, first attempts to artificially induce headache and develop an experimental human model of migraine began using intravenous nitroglycerin (NTG), another potent vasodilator [25]. NTG administration provoked an initial mild headache in healthy volunteers and a delayed headache with migrainous features in patients with migraine [25], and it was later found that CGRP levels in peripheral vasculature were increased after such induction [26]. Its association with migraine was proved in human studies, where short-term CGRP elevation in local vasculature was observed in patient blood during migraine attacks with and without aura [27]. CGRP levels after migraine attacks were also found to be decreased with sumatriptan treatment and coinciding with headache improvement [28], providing evidence of this neuropeptide being involved in the headache phase of migraine. Further trials led to CGRP being injected into the peripheral vasculature of migraine patients causing a delayed headache with migrainous features, confirming that CGRP plays a key role in migraine pathophysiology [29]. Consequently, novel drugs were developed to target CGRP signaling through either direct blockade of CGRP or its receptor.

## Pharmacology

**Fig. 2:**
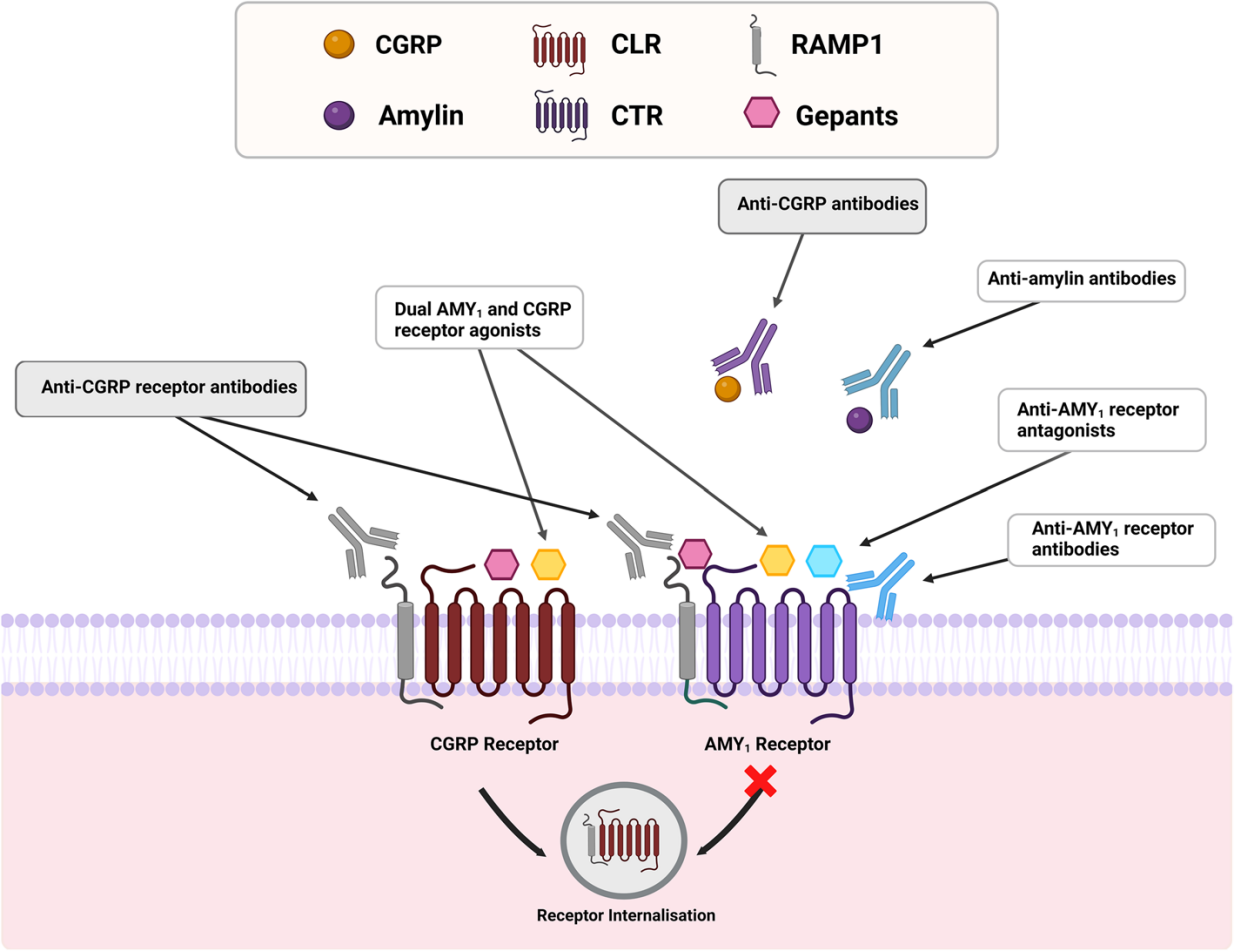
Crosstalk between CGRP- and Amylin-based therapies. CGRP and amylin 1 (AMY_1_) receptors are formed by association of either CLR or CTR with RAMP1, respectively. CGRP and amylin are equipotent at the AMY_1_ receptor, while CGRP is more potent at the canonical CGRP receptor. These receptors have a distinct internalization profile. Current antimigraine drugs targeting CGRP (blue boxes) and potential antimigraine amylin drugs (white boxes) are shown. DACRAs, dual amylin and calcitonin receptor antagonist. Taken and modified from [31, 32]

CGRP is a member of the calcitonin (CT)/CGRP family of neuropeptides which also includes CT, amylin, adrenomedullin and intermedin/adrenomedullin 2 [7], with CGRP and amylin being the most closely-related in terms of amino acid sequence and function [30]. The receptors that bind CGRP have only recently been fully characterized [7]. As shown in Fig. 2, the canonical CGRP receptor is atypical among G-protein-coupled receptors, as its functionality depends on the presence of a G-protein coupled calcitonin receptor-like receptor (CLR), a receptor activity-modifying protein 1 (RAMP1), and the receptor component protein (RCP) [31, 32]. The ligand-binding domain of the CGRP receptor is located at the extracellular domain and transmembrane bundle of CLR, with no direct involvement of RAMP1 but acting allosterically to enable CGRP recognition [7, 33]. Moreover, the CLR:RAMP1 complex allows reaching the plasma membrane and binding CGRP with high affinity [34]. Thus, co-expression of CLR and RAMP1 is necessary for CGRP to bind to the canonical CGRP receptor [35].

Moreover, CGRP is equipotent at activating a second receptor, the amylin 1 (AMY_1_) receptor, which contains RAMP1 but is coupled with the CT receptor (CTR) [31, 36]. The activation of each of these receptors causes an increase in cAMP levels with downstream activation of protein kinase A, suggesting they are of the Gαs-coupled type [7]. Interestingly, in contrast to the CGRP receptor, the AMY_1_ receptor undergoes scarce internalization (Fig. 2) [31]. As both receptors seem to colocalize in trigeminal fibers [37], there could be relevant feedback loops between CGRP acting at the CGRP and and AMY_1_ receptors [37, 38]. However, it remains to be determined how this contributes to CGRP physiology and the effectiveness of current anti-CGRP drugs.

It is well-established that CGRP (peptide, receptors, and mRNA) is diffusely expressed across the peripheral and CNS in a variety of cell types [35, 39]. Understanding where CGRP and its receptors are expressed is relevant to understanding the mechanisms of drugs which target this neuropeptide system as well as drug-drug interactions and potential adverse effects.

As shown in Fig. 1, the main sources of CGRP release are from trigeminal afferents [40, 41], that originate in the trigeminal ganglion and which upon electrical, chemical, or mechanical stimulation or during spontaneous migraine attacks release CGRP, leading to dysfunctional nociceptive transmission and eventually headache [27, 28]. Furthermore, recent immunofluorescence studies of these fibers have revealed that CGRP is mainly localized in C-fibers, whereas the components of the CGRP receptor (CLR:RAMP1) are predominantly found in Aδ-fibers [42, 43]. This suggests that local release of CGRP from trigeminal C-fibers activates CGRP receptors in Aδ-fibers and adjacent cells [43, 44].

Centrally, CGRP and its receptor have been shown to be highly expressed in the amygdala, locus coeruleus, striatum, hypothalamus, and parabrachial nucleus [7, 35, 45]. Studies in primates have also shown RAMP1 and CLR mRNA expression in the pineal gland [39]. Interestingly, Purkinje cell bodies in the cerebellum colocalize CGRP and CGRP receptor (CLR and RAMP1) subunits [46], which also suggests that autocrine signaling may occur. As the complexity of the CGRPergic system and the interactions with its family of peptides is yet to be understood, a key question remains highly contested: where is the main antiheadache site of action (peripheral vs central) of anti-CGRP therapies? Thus, the following sections will provide a critical analysis of the current evidence of the likely mode and site of action of CGRP antagonists and antibodies, as well as antimigraine drugs that indirectly modulate CGRP.

## Gepants

Due to the key role of CGRP in migraine pathophysiology, selective small-molecule CGRP receptor antagonists (gepants) were synthesized and proved to be effective in the acute [47, 48] and preventive treatment of migraine [49, 50]. Although the first generation of gepants (e.g., olcegepant and telcagepant) was promising, pharmacokinetic and hepatotoxicity limitations stopped their development [51]. However, a new generation of gepants was developed and overall, all have shown efficacy and safety profiles with no demonstrable abnormalities in serum transaminases (reviewed in [52]). As shown in Table 1, ubrogepant, rimegepant and zavegepant (the first intranasal gepant) are effective for the acute treatment of migraine [52–54], whereas atogepant and rimegepant have demonstrated to be safe, efficacious and tolerable as a preventive treatment for migraine [49, 50].

**Table 1:** Gepants currently available

	**Ubrogepant**	**Zavegepant**	**Rimegepant**	**Atogepant**
Indication	Acute treatment of migraine with or without aura in adults	Acute treatment of migraine with or without aura in adults	Acute treatment of migraine with or without aura in adults Preventive treatment of episodic migraine in adults	Preventive treatment of episodic migraine in adults
Dosage	50–100 mg p/o as needed. Second dose 2 h after the initial dose if needed. Max 200 mg/24 h No established safety of treating > 8 migraines / 30 days	10 mg intranasal No established safety of treating > 8 migraine / 30 days	75 mg p/o as needed. Max 75 mg /25 h No established safety of using > 18 doses /30 days	10 mg, 30 mg or 60 mg p/o daily Safe for daily use

### Mode of action

Gepants bind with high affinity to the canonical CGRP receptor (CLR:RAMP1), and they seem to have negligible to low affinity for adrenomedullin receptors, composed of RAMP2 and RAMP3 proteins [36, 55]. However, as shown in Fig. 2, the AMY_1_ receptor (CTR:RAMP1), one of three amylin receptors, could also be targeted by CGRP receptor antagonists. This is explained by the antagonist selectivity driven by the RAMP receptor subunit and the similar RAMP1 subunit shared by the CGRP and AMY_1_ receptors [36]. Illustrating this cross-reactivity, the antagonist selectivity of rimegepant is only 30-fold higher at CGRP receptors than AMY_1_ receptors [56]. The antagonism at these two receptors is a hypothesis proposed to explain the dual inhibition of both C-fibers and Aδ-fibers by atogepant observed in the trigeminal ganglion of rats [57], however the clinical implications of blocking both receptors remain to be determined.

### Site of action

Based on their small molecular weight, the gepants could theoretically cross the BBB [51] hence, it would be expected that their antimigraine efficacy is the result of antagonizing the CGRP receptor both peripherally and centrally. In this regard, electrophysiological studies in rats have revealed that the CNS structures being targeted by intravenous or intraperitoneal administration of gepants include second- and third-order nociceptive trigeminovascular neurons [58, 59], as well as the periaqueductal gray and nucleus raphe magnus, descending pain modulatory systems [60, 61]. These drugs were also able to ameliorate cortical spreading depression (CSD)-induced photophobia and hypomobility in mice [62]. However, the placement of recording electrodes or direct delivery of drugs might break the BBB and contribute to the observed effects of gepants. Moreover, positron emission tomography (PET) studies in non-human primates with the CGRP receptor tracer [C-11]MK-4322 and telcagepant indicate that gepants do not require to penetrate the BBB to exert their antimigraine action.

Sur et al. found that after the oral administration of telcagepant only a small percentage could be detected in cerebrospinal fluid (CSF) as compared to plasma (CSF/plasma ratio of ~ 1%) [63]. Furthermore, another study in primate and human brain regions revealed that only supratherapeutic doses of telcagepant were able to achieve a moderate CGRP receptor occupancy (43–58%), while in healthy volunteers clinically-relevant doses only achieved low receptor occupancy (≤ 10%) [64]. Lastly, another study with the same PET tracer found no evidence of CGRP receptor central occupancy after therapeutic doses of telcagepant in migraine patients during ictal and interictal periods [65]. Taken together, these results suggest that at therapeutic concentrations, a central antagonism of the CGRP receptor is probably not required for the efficacy of gepants in migraine treatment. Moreover, gepants have a very limited ability to cross the BBB [66], and it remains to be determined whether they can target CNS areas that are not covered by the BBB (i.e., circumventricular organs), where dense CGRP and amylin binding is present [67, 68], and the clinical relevance, if any.

## Anti-CGRP monoclonal antibodies

Compared to gepants, monoclonal antibodies (mAbs) are large heterodimeric protein molecules (molecular weight ~ 150 kDa) designed to block targeted molecules, especially for therapeutic purposes (Table 2). Four monoclonal antibodies have been developed to target CGRP signaling so far. Erenumab acts over the CGRP receptor, whereas fremanezumab, galcanezumab and eptinezumab target the CGRP ligand itself. These four drugs have demonstrated to be safe, efficacious, and tolerable as a preventive treatment for migraine (galcanezumab also for episodic cluster headache prevention) and are currently studied in other headache disorders. Table 3 summarizes their characteristics and current clinical use. As these drugs have been mainly investigated in migraine, this section will focus on the evidence on this primary headache disorder.

**Table 2:** Molecular characteristics of anti-CGRP monoclonal antibodies and gepants. Adapted from [64, 69–71]

	Gepants	Anti-CGRP monoclonal antibodies
Target	CGRP receptor	CGRP receptor or ligand
Clearance	Liver, kidney	Reticuloendothelial system
Half-life	5–11 h	3–7 weeks
Size	0.5–0.6 kDa	143–146 kDa
Ability to cross blood–brain barrier	Low (1.4% CSF/plasma ratio)	No
Administration	Oral, intranasal	Parenteral
Immunogenicity	No	Yes

**Table 3:** Anti-CGRP monoclonal antibodies

**Monoclonal antibodies**	**Erenumab**	**Galcanezumab**	**Fremanezumab**	**Eptinezumab**
**Target**	CGRP receptor	CGRP ligand	CGRP ligand	CGRP ligand
**IgG type**	IgG2, human	IgG4, humanized	IgG2a, humanized	IgG1, humanized
**Administration**	Monthly SC	Monthly SC	Monthly or quarterly SC	Quarterly IV
**Doses approved for migraine prevention (EM, CM)**	70 mg or 140 mg	120 mg (240 mg loading dose)	225 mg monthly or 675 mg quarterly	100 mg or 300 mg
**Other headache disorders** (approved or under investigation)	cCH, PTH	eCH (approved 300 mg monthly), cCH (no primary endpoint met)	eCH and cCH (primary endpoint unlikely to be met), PTH	eCH, cCH

*Abbreviations*: *cCH* Chronic cluster headache, *eCH* Episodic cluster headache, *IV* Intravenous, *SC* Subcutaneous injection, *PTH* Acute post-traumatic headache

### Mode of action

Erenumab binds to the CLR:RAMP1 extracellular domain interface of the CGRP receptor to block it [72]. However, the binding of erenumab to the CGRP receptor also prevents the action of other two peptides, adrenomedullin and intermedin/adrenomedullin 2, that are also able to act on the CGRP receptor [55]. Erenumab is highly selective for the canonical CGRP receptor (CLR:RAMP1) but can still bind to other receptors with less affinity such as CLR:RAMP2 and CLR:RAMP3 (adrenomedullin receptors), or CTR:RAMP1 (AMY_1_ receptor) complexes [73]. This is relevant as CGRP is able to activate these other receptors, especially AMY_1_ [74]. Moreover, genetic variants occurring at the CGRP receptor may influence the peptide and/or drug affinity response [75]. In the case of galcanezumab, fremanezumab and eptinezumab, these monoclonal antibodies bind the same region of CGRP ligand that binds to the receptor, thus rendering both α-CGRP and β-CGRP incapable of binding to the CGRP receptor [76, 77]. Because of this ligand-specific mechanism, there is no evidence that fremanezumab interacts with the AMY_1_ receptor, therefore not affecting amylin responses [55].

Both CGRP ligand or receptor mechanisms interrupt CGRP-induced signaling via cAMP accumulation and potentially inhibit the CGRP receptor internalization [78]. However, mechanisms of receptor internalization are complex and the drugs themselves can be internalized. Erenumab seems to undergo internalization in CGRP and AMY_1_ receptor expressing cells, interestingly this was not the case for fremanezumab [55]. Gepants also undergo internalization and due to their different pharmacokinetic profile, they may also block CGRP signaling from within endosomes [79], this could explain the effective use of gepants for acute treatment during concomitant erenumab preventive administration [80]. The meaning and clinical significance of internalization and intracellular signaling as molecular mechanisms remain unknown.

Serum levels of CGRP after treatment have been explored, especially with erenumab, to assess the presence of CGRP up or down-regulation mechanisms induced by the treatment [81–83]. Results are still highly controversial and this is mainly due to the different methodologies used, however few studies showed reduction in CGRP levels after treatment [83] or no difference [81, 82], questioning the role of using CGRP as a potential biomarker.

Recently, new molecular mechanisms have been disclosed and it has been demonstrated that CGRP released from trigeminal fibers can signal on surrounding Schwann cells [21] and can be taken up and re-released in the dura. However, the latter mechanism seems not to be mediated by presynaptic CGRP receptors, as CGRP receptor antagonists were not able to block the uptake of CGRP. Such mechanisms may be relevant in regulating CGRP availability and may also influence mAbs and gepants treatment responses [84].

### Site of action

The exact site of action of anti-CGRP mAbs in migraine prevention is only partially understood. As previously mentioned, CGRP acts as a vasodilator at the level of the vascular smooth-muscle cells of intracranial arteries as well as a nociceptive neuropeptide in perivascular trigeminal primary afferents [85]. Thus, the probable preventive effect of anti-CGRP mAbs in migraine is mediated by inhibition of first-order trigeminovascular neurons that are involved in pain transmission. Fremanezumab is known to block CGRP-induced vasodilation in human meningeal arteries in vitro [86]. Moreover, preclinical data have shown that, in a migraine rat model of CSD, fremanezumab inhibited Aδ- but not C-type primary afferent meningeal nociceptors, that innervate the cranial dura [44]. Aδ-fibers are activated by release of CGRP from C-type nociceptors after CSD and activate specific type of central trigeminovascular neurons, the high-threshold (HT) neurons, whose input is predominantly from Aδ-fibers and eventually transmit the nociceptive signals to the thalamus [44]. Another study, confirmed that fremanezumab selectively inhibited the activation and sensitization of HT neurons in a rat model of CSD-evoked or mechanical stimulation of the dura [87]. These data remark that the activation of the Aδ-HT nociceptive pathway may be sufficient for the initiation of headache perception and the development of central sensitization and its clinical correlate that is allodynia [44, 87].

Anti-CGRP mAbs are thought to act mainly peripherally, due to their large size. Two studies using radiolabeled mAbs confirmed this hypothesis [20, 69]. In rats with uncompromised BBB, fremanezumab could be detected in the dura, dural blood vessels, trigeminal ganglion, C2 dorsal root ganglion, the parasympathetic sphenopalatine ganglion, and the sympathetic superior cervical ganglion but not in central areas such as the cortex, spinal trigeminal nucleus, thalamus, nor the hypothalamus where the BBB is relatively open [20]. However, all the previous mentioned studies support the concept that by acting in the periphery, the anti-CGRP mAbs also exert a modulation of central neurons, which probably contributes to their preventive effect. This is also observed clinically, where central anticipatory and accompanying symptoms of the headache phase seem to improve with anti-CGRP mAbs treatment [88]. Moreover, translational studies using EEG techniques have shown that abnormal visual cortical activity can be restored with galcanezumab [89] and a study using functional MRI showed that galcanezumab decreases hypothalamic activation [90].

A final remark on the site of action of anti-CGRP mAbs must be done in relation to headache disorders other than migraine. Melo-Carrillo et al. observed that fremanezumab was not able to inhibit the activation of HT neurons from mechanical stimulation of other regions such as the skin or cornea [87], thus suggesting a selectivity of these drugs to migraine, but not to other cranial and/or extracranial pain conditions, such as trigeminal neuralgia. A clinical study has been conducted on erenumab in trigeminal neuralgia with negative results [91]. However, CGRP mechanisms can indeed be present and therefore be targeted by anti-CGRP mAbs in other headache disorders. Galcanezumab is a Food and Drug Administration approved treatment for episodic cluster headache (CH) [92], but the phase 3 randomized controlled trial (RCT) on chronic CH did not meet the primary endpoint [93]. Nevertheless, in clinical practice these latter patients also seem to benefit from the treatment [94]. Eptinezumab and erenumab are currently being studied in episodic and/or chronic CH [95–97], whereas studies on fremanezumab in both episodic and chronic CH were terminated following a futility analysis which revealed that primary outcomes were unlikely to be met [98, 99]. Among secondary headache disorders, post-traumatic headache [45, 46] involves CGRP [100, 101], with promising preliminary studies [102], although RCTs on anti-CGRP mAbs are still ongoing [103, 104]. Moreover, the role of CGRP and anti-CGRP mAbs is being investigated in headache attributed to idiopathic intracranial hypertension [105, 106].

## Antimigraine drugs that modulate CGRP

### 5-HT_1_ agonists

In the last decades, the gold standard for acute migraine treatment has been the triptans, 5-HT_1B/1D/(1F)_ receptor agonists. During a migraine attack, they can normalize the elevated CGRP plasma levels by inhibiting further release from trigeminal afferents, thereby decreasing nociceptive transmission (Fig. 1). Experimentally, triptans inhibit CGRP release from peripheral and central trigeminal fibers, however, low lipophilicity and interactions with BBB efflux transporters limit their central actions in vivo [107]. In addition, activation of a high population of 5-HT_1B_ receptors on vascular smooth muscle is mainly associated with potentially dangerous cardiovascular side effects, contraindicating its use in patients with heart disease and hypertension [108, 109].

Ditans are a new group of acute antimigraine drugs which are highly lipophilic and selective for the 5-HT_1F_ receptor [110]. Lasmiditan is the first drug approved in this class. Mechanistically, ditans inhibit the release of CGRP from peripheral and central trigeminal terminals [111]. Higher incidence of CNS-related adverse effects like dizziness, paresthesia, vertigo, fatigue, and somnolence can be due to the high BBB permeability and abundant expression of 5-HT_1F_ receptor in cortical areas, hippocampal formation, and claustrum as well as throughout the vestibular system [112, 113]. Ditans lack cardiovascular side effects [114], which may offer an alternative to triptans in patients with cardiovascular diseases; however, odds ratio for pain freedom and pain relief at 2 h were lower when compared with most triptans [115].

### Preventive drugs

Although the key mechanisms and sites of action for medications used in migraine prevention remain unclear, it seems that almost all affect the trigeminal CGRP system indirectly [116, 117]. They inhibit CGRP release and consequently reverse sensitization in chronic migraine [116, 118]. Therefore, CGRP reduction might be at play in determining the effectiveness of non-specific anti-migraine preventive drugs, whereas interictal CGRP levels can be a predictor of response to these preventives [118, 119].

Topiramate is an antiepileptic drug that can be efficacious in migraine prevention via different mechanisms [116]. Preclinical studies demonstrated that it decreases CGRP release from sensory trigeminal neurons in response to depolarizing stimuli, in a time-concentration manner, hence, decreasing nociception [120]. Topiramate also inhibits nitric oxide/proton mediated CGRP release from peripheral afferents [120]. However, unaltered CGRP plasma levels by a low-dose of topiramate in a small clinical trial indicates that the antimigraine effects of topiramate could be independent from the CGRP pathway [121].

The other non-specific antimigraine medication which acts by inhibiting CGRP release from meningeal and extracranial thin myelinated C-fibers is onabotulinumtoxinA (BoNT-A) [122], an injectable formulation of a neurotoxin derived from the bacterium clostridium botulinum. The first mechanism through which BoNT-A blocks CGRP release is by preventing the adhesion of synaptic vesicles to the cell surface [123]. Furthermore, BoNT-A administration might reduce transient receptor potential (TRP) channel expression, particularly TRP vanilloid 1 (TRPV1) and TRP ankyrin1 (TRPA1), on unmyelinated C-fibers in the synaptic membrane and as a result decrease response to nociceptive stimuli and CGRP release (Fig. 1) [123, 124]. Therefore, BoNT-A can revert and prevent CGRP-dependent activation of thick myelinated Aδ-nociceptors, meningeal vessels, and immune cells [122, 125, 126].

Propranolol is a non-selective β-adrenoceptor antagonist also frequently used to prevent migraine attacks. However, it has been shown that it has agonist effects on prejunctional 5-HT_1D_ and 5-HT_1F_ receptors in trigeminal fibers through which can inhibit the release of CGRP from these fibers that innervate the forehead skin and dura mater [117]. Interestingly, a single point mutation in the seventh transmembrane domain of the 5-HT_1_ receptor increases 100–1000 fold the affinity of β-adrenoreceptor antagonists for the 5-HT_1D/1F_ receptors [127].

Valproate is widely used as treatment for seizure and bipolar disorder. It also prevents migraine attacks through different mechanisms. Animal studies have shown that it restores brain GABA levels [128], hence neuron activation inhibition [129], that might affect CGRP and c-fos expression via central/peripheral sites of action [130]. Valproate also inhibits NF-kB pathway in the TNC, leading to a reduction in CGRP synthesis [130].

It is remarkable that not only non-CGRP preventive drugs, but also specific medications used for acute migraine attacks can modulate the CGRP pathway. This finding can improve our understanding of migraine pathophysiology and be of clinical interest to determine more efficacious therapeutic strategies.

## Non-responders to CGRP-targeted therapies in migraine

The existence of non-responders to anti-CGRP mAbs or gepants is an interesting and complex matter. Several explanations are possible. First, a greater CGRP antagonism at a central level may be necessary for migraine prevention. In this context, future studies comparing directly gepants, that are potentially able to act centrally, and anti-CGRP mAbs may provide insights on this matter. However, at present, clinical trials for each drug report similar responder rates [70, 131] and preclinical data only suggest that, in rat models, anti-CGRP monoclonal antibodies may have, as expected, a longer duration of effect but also a more rapid onset of response [132]. Second, anti-CGRP antagonism may be insufficient due to the existence of concomitant other pathophysiological pathways. CSD, for example, is able to activate C-fiber meningeal nociceptors that eventually activate another type of central trigeminovascular neurons, the wide-dynamic range (WDR) neurons [43, 133]. The absence of CGRP receptors from the meningeal C-fibers renders the C-WDR pathway CGRP-independent, and confirms why in preclinical studies it is unresponsive to fremanezumab [44]. Nevertheless, C-fibers and, consequently, WDR neurons can be inhibited by administration of BoNT-A [134], providing a rationale for associating BoNT-A to anti-CGRP mAbs in clinical practice [124]. Clinical studies are still scarce but have shown potential benefits of combination therapy [125, 135]. Third, non-responders could have a state of central neuron sensitization, supported by the presence of non-ictal allodynia, that is independent from peripheral activation and that cannot be attenuated by anti-CGRP mAbs [136]. In clinical practice, presence of non-ictal allodynia seems to be a useful predictor of lack of response to galcanezumab [136]. Finally, not only molecular mechanisms, as previously mentioned, but also the mode of action may differ from one anti-CGRP mAb to another, influencing treatment response. One fMRI study showed differences between erenumab and galcanezumab in the brain areas with decreased activity after treatment [90]. Although its full meaning is unclear, this finding could still have implications on therapeutic outcomes of anti-CGRP mAbs and potentially further supports switching non-responders to another anti-CGRP mAb (or gepants approved for preventive treatment) as a therapeutic option in clinical practice.

## Combining and/or switching drugs that modulate CGRP

It is logical to think that different molecular mechanisms may result in different response rates, tolerability, and side effects. However, at present we lack a real comparative study among anti-CGRP mAbs (or gepants used for preventive treatment) and response rates are at least similar in clinical trials and real-world studies [70, 137–139]. Yet, there are indirect clinical signs pointing to different mechanisms. First, side effects are different, and specifically constipation has been described for erenumab, galcanezumab and atogepant [52, 140]. This may be because these drugs can simultaneously block the CGRP and AMY_1_ receptors that may be more relevant at gastro-intestinal level [141]. Second, in clinical practice there are patients not responding to one anti-CGRP mAb that are still able to respond to another with a different mechanism [142]. The different mechanistic and clinical scenarios are described in Table 4.

**Table 4:** Mechanistic and clinical scenarios of anti-CGRP mAbs

**(Super-) Responders to mAbs**	**Non-responder to erenumab**		**Non-responder to anti-CGRP ligand mAbs**	
	**Responder to switch (ligand)**	**Non-responder to switch (ligand)**	**Responder to switch (erenumab)**	**Non-responder to switch (erenumab)**
**Molecular mechanism**				
CGRP antagonism sufficient	CGRP antagonism sufficient but: 1. CGRP receptor block insufficient 2. CGRP must be blocked elsewhere	CGRP antagonism insufficiently blocked and/or other molecules may be involved	CGRP antagonism sufficient but: 1. circulating CGRP block insufficient 2. other molecules acting on the CGRP receptor or similar receptor (AMY_1_) must be blocked	CGRP antagonism insufficiently blocked and/or other molecules may be involved
**Mode of Action**				
Peripheral CGRP antagonism is sufficient	Peripheral CGRP antagonism is sufficient	Peripheral CGRP antagonism is probably insufficient and: 1. More central action may be required 2. Other CGRP-independent pathways are involved peripherally or centrally	Peripheral CGRP antagonism is sufficient	Peripheral CGRP antagonism is probably insufficient and: 1. More central action may be required 2. Other CGRP-independent pathways are involved peripherally or centrally

As some studies are starting to show that combining gepants and triptans [143, 144], or gepants with anti-CGRP mAbs seem safe, well-tolerated and could have synergistic effects on pain relief in patients with migraine [80, 145], a combined antagonism of CGRP may give wider options to clinicians to choose between medications from different classes based on individual patient’s risks and responses Concurrently, the involvement of amylin in migraine pathophysiology is becoming evident [146, 147], which suggests that development of novel drugs targeting the AMY_1_ receptor, either via selective antagonists or antibodies, might also be effective for treating migraine (Fig. 2). Obviously, further clinical studies are warranted to evaluate the safety and efficacy of dual blockage of CGRP (or CGRP and amylin), due to its diverse physiological functions in the human body.

## Other sites of action

As CGRP exerts different physiological functions, CGRP antagonism through anti-CGRP mAbs or gepants may mediate different (side)-effects; it is also worth considering that these drugs might have additional non-sensory antinociceptive sites of action, as resident immune cells, fibroblasts, and dural vessels are capable of modulating the activity of meningeal nociceptors [12, 40]. Clinical studies have demonstrated that these drugs are generally safe and well-tolerated, even at long term [70, 148]. However, concerns on cardiovascular safety have been raised, specifically due to the vasodilating properties of CGRP and its potential protective role during cardiac and/or cerebral ischemia [149]. A preclinical study showed that, although erenumab inhibits the vasodilatory responses of CGRP especially in the distal portion of the human coronary artery, it does not influence those of other vasodilators [150]. A clinical study on erenumab in patients with stable angina showed no significant changes in exercise treadmill test [151], supporting safety in this population. However, further studies assessing cardiovascular safety of anti-CGRP mAbs and gepants should be conducted, specifically focusing on women population that may be more prone to cardiac events involving the distal portion of the coronary artery [152]. Other effects on the vascular system may be responsible for reported cases of Raynaud syndrome in patients treated with mAbs, but data are still unclear and the studies are warranted [153].

Among other sites of action of anti-CGRP mAbs and gepants, data from real-world experience of these drugs have disclosed that the GI can be affected, resulting as previously mentioned, in constipation [52, 141]. These drugs may also block the role of CGRP in hair growth, leading to alopecia [154], and bone formation [155] all these potential effects need to be better investigated.

## Conclusion

Drugs that block the trigeminal CGRPergic system are effective in the preventive and acute treatment of migraine. Current lines of evidence indicate that the therapeutic effect of the current anti-CGRP mAbs is mainly peripheral, and this also appears to apply for gepants. Even though gepants could cross the BBB, different studies indicate that this site does not appear to play a prominent role in the antimigraine effects of these drugs. So, all this reveals that migraine attacks can be treated and prevented via peripheral blockage of CGRP. Further research is clearly needed to fully elucidate the pharmacology of anti-CGRP therapies, this could allow us to understand why some patients with migraine are non-responders or stop responding to these medications.

## Data Availability

All included references in the present review article are available on the Internet.
